# Increasing hospitalisation of patients with herpes zoster ophthalmicus—an interdisciplinary retrospective analysis

**DOI:** 10.1007/s00417-023-06277-w

**Published:** 2023-10-20

**Authors:** Rebecca Diehl, Cornelius Wiedenmann, Thomas Reinhard, Daniel Böhringer, Franziska Schauer

**Affiliations:** 1 Department of Dermatology, Medical Center, Faculty of Medicine, University of Freiburg, Freiburg, Germany; 2 Eye Center, Medical Center, Faculty of Medicine, University of Freiburg, Freiburg, Germany

**Keywords:** Herpes zoster ophthalmicus, Vaccination, Herpes zoster, Chickenpox

## Abstract

**Background:**

The occurrence of herpes zoster is rising globally. Future trends will be influenced by changes in population demographics and the growing number of patients at risk. Overall this poses a challenge for healthcare systems.

**Methods:**

In our interdisciplinary, single-centre retrospective analysis, we aimed to assess the burden of the disease within the Department of Dermatology and the Eye Centre from the Medical Centre, University of Freiburg from 2009—2022. We obtained data from 3034 cases coded using the ICD-10 B02.x. Patients were characterised by sex, age, year of treatment, and type of treatment (inpatient vs. outpatient).

**Results:**

Overall we observed a 200% increase in the number of herpes zoster patients over the 13-year period. Upon closer analysis, this was mainly due to a rise in inpatient treatment for herpes zoster ophthalmicus.

**Conclusions:**

If the incidence of herpes zoster ophthalmicus continues to increase at the current rate the number of hospitalisations of zoster ophthalmicus would double by 2040, assuming guideline-appropriate treatment. Overall, the results show a growing need for inpatient ophthalmological care.

**Supplementary information:**

The online version contains supplementary material available at 10.1007/s00417-023-06277-w.


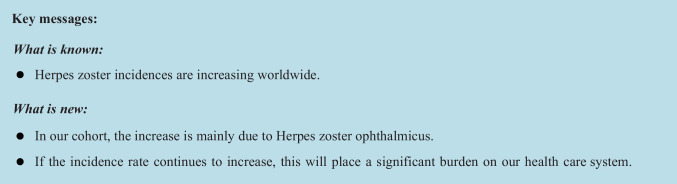


## Introduction

The incidence of herpes zoster (HZ) has been increasing in many parts of the world [1, 2]. This concerning trend warrants a better understanding of the factors driving increased hospitalisation of HZ patients.

Since 2004 varicella vaccination is recommended for most children of age 9 months to 18 years by the Standing Committee on vaccination (STIKO) in Germany. Several mono and combi attenuated vaccines have been made available since September 2003 [3]. The HZ vaccine Shingrix, an inactivated vaccine, was approved by the European Medicines Agency (EMA) in March 2018, and in Germany, since December 2018, the STIKO recommended vaccination for people aged 60 years and older and for people aged 50 years and older with an increased risk of HZ and postherpetic neuralgia (PHN) [4]. The introduction of these vaccines was expected to affect the incidence of primary varicella infections as well as reactivation.

Herpes zoster ophthalmicus (HZO) refers to shingles of the ophthalmic division of the trigeminal nerve (V1). HZO can have potentially devastating ophthalmic complications including keratitis, uveitis, glaucoma, optic neuropathy and acute retinal necrosis (ARN) as well as non-ophthalmic complications such as PHN, secondary bacterial infections, encephalitis and vasculopathy [5–11]. Prompt treatment with antivirals and close monitoring are required to minimise the risk of vision loss and other sequelae [12, 13]. After guideline revision in 2018, intravenous (i.v.) aciclovir therapy for herpes zoster in the head and neck region including HZO was promoted. The recommendation is based on expert consensus [14].

In this study, we analyse data from the Department of Dermatology and the Eye Centre of the University Medical Centre Freiburg from 2009 to 2022 to evaluate the development of HZ(O) incidences and to characterise the demographic and clinical profiles of all HZ patients. The findings from this study will help inform strategies to optimize management of HZ in the future.

## Methods

Anonymous data of all patients who were treated for the time period 2009/01/01 to 2022/12/31 for ICD B02.0–9 were retrospectively accessed at the Department of Dermatology and Eye Center from the Medical Center, University of Freiburg from 2009—2022. The recorded data included ICD-10 code, year, sex, age, treatment site (dermatology vs. ophthalmology vs. both) and patients’ postal code at time of treatment. Statistical analyses were conducted using the R software version 4.2.3 (2023–03-15) and R Studio version 2023.03.0 + 386. P-values below 0.05 were considered statistically significant.

## Results

In total, we were able to evaluate 3034 treated herpes zoster patients from 2009—2022. 1879 patients were treated in the department of dermatology, 920 cases in the eye centre and 235 patients presented in both departments. The median age was 64 years [q1: 49, q3: 76]. Overall, there were more female patients with 1586 and fewer male patients with 1448. Affected women had a median age of 66 years [q1: 51, q3: 77], which is older than affected men with median age of 63 years [q1: 46, q3: 75]. From 2009 to 2022, there was an approximately eightfold increase in the number of HZO inpatients in the eye centre (Supplement Fig. 1a). In contrast, no increase in the number of dermatologic inpatients was observed (Supplement Fig. 1b). For outpatients, we observed the opposite: Outpatients decreased during the investigated time period in the eye centre, while there were increasing numbers in the department of dermatology (Supplement Fig. 1b).

**Fig. 1:**
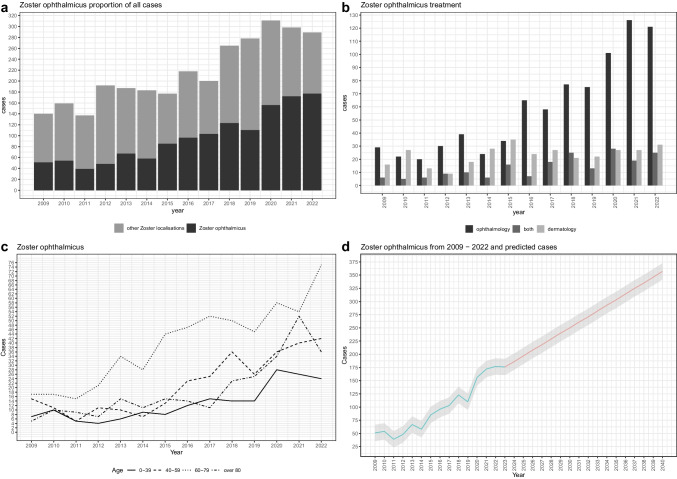
**a**: Share of HZO patients in total HZ patients at both hospitals. **b**: HZO treatment according to speciality. **c**: HZO patients sorted by age from 2009 - 2022, **d**: HZO patients at both hospitals from 2009 to 2022 and predicted cases assumed at stable growth

**Fig. 2:**
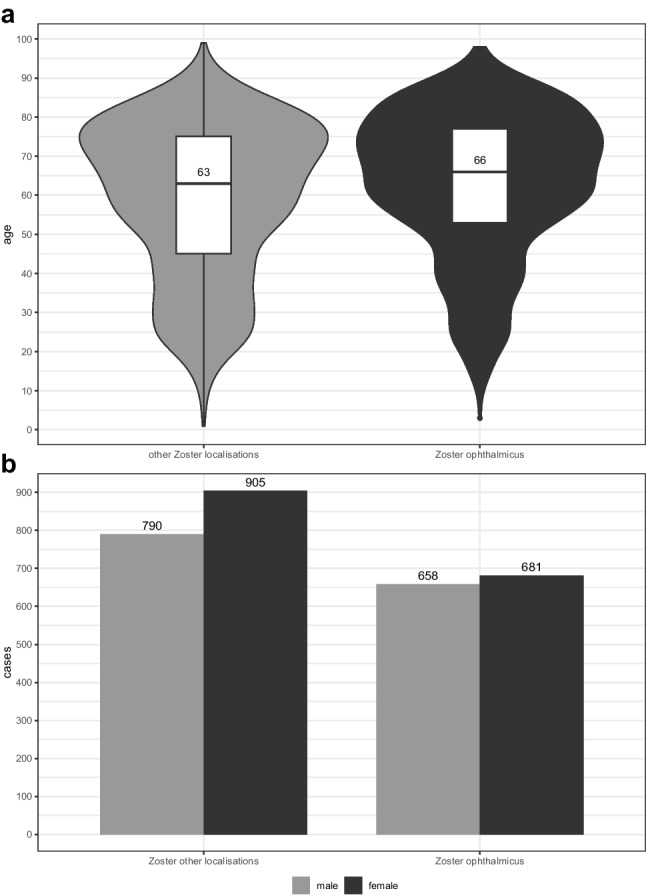
**a**: Age distribution with median of patients with HZO and HZ of other localisations. **b**: Gender distribution HZ other localisations and HZO

The overall increase in HZ patients is mainly due to the increase in HZO patients (Fig. 1a), which were predominantly treated at the eye centre (Fig. 1b). Overall, the proportion of HZO patients per year increases significantly compared to the total number of patients treated at the eye clinic (p < 0,05). A shift in patients from dermatology to ophthalmology seems unlikely, as HZO patients have remained consistently treated in the eye centre over the years (Fig. 1b). Patients with HZO had a higher mean age of 66 years than patients with HZ at other sites (Fig. 2a). Regardless of the localisation of HZ, men and women are affected about equally often (Fig. 2b). Looking at the age groups of 0–40, 40–59, 60–79, and 80 years and over, an increase in HZO patients was observed in all age groups (p < 0,05). The age group most affected was 60–79 years (Fig. 1c). In comparison, the numbers for HZ in localisations other than the eye remained relatively constant across all age groups. There was only a slight increase (*p* < 0,05) observed in the age groups of 0–40, 40–59, and 80 years and over. Interestingly, there was a decline in the number of affected patients within the 60–79 age group since 2018 (*p* < 0.05, Supplement Fig. 1c). Eye centre patients came from a wider catchment area than the dermatology patients (Supplement Fig. 1d). When calculating the linear regression of HZO patients per year, a highly significant correlation is observed (*p* < 0.05) and variance of 0.9016. Assuming that the increase in HZO patients remains constant, one can expect a doubling of HZO cases by 2040 (Fig. 1d).

## Discussion

The incidence of HZ has been increasing steadily in Germany and worldwide over the past 15 years [1, 2, 15]. Our single centre study also reflects this trend. The reasons for this increase are widely debatated.

The impact of varicella vaccination HZ incidence remains controversial. Since introducing the varicella vaccine in Germany in 2009, varicella infections have significantly decreased [16]. This reduces endogenous boosting due to less exposure to the varicella zoster virus [17]. Consequently, cell-mediated immunity may decrease, potentially causing higher HZ rates in adults [17]. However, HZ incidence was already rising before the varicella vaccine, some studies consider the increase independent of varicella vaccination [18].

An attenuated live vaccine for HZ was approved in 2006 and became available in Germany in 2013 [19]. However, the permanent vaccination commission of the Robert Koch Institute (RKI) in Germany does not recommend it as a standard vaccine until. The reasons include its low efficacy and the inability to vaccinate vulnerable patient groups with congenital or acquired immunodeficiency [20]. There is no data on the vaccination rate with this attenuated live vaccine. Given the RKI's missing recommendation and its low effectiveness, its impact on HZ incidence is expected to be minimal. Since 2018, the Robert Koch Institute (RKI) has recommended HZ vaccination with the inactivated vaccine in Germany for those 60 and older [21]. For patients at risk, vaccination is already recommended at the age of 50 [21]. Full vaccination protection is obtained by vaccinating twice with an interval of 2–4 months. When fully vaccinated, the overall risk of HZ decreases by 92% [22, 23]. Protection wanes in older patients [23]. In our data, we see a decrease in the number of HZ patients from other locations since 2018 in the 60–79 age group, which could be a first indication of building vaccination protection. However, HZO patients in this age group are increasing. In Germany, the initial HZ vaccination rate is 11.5% of eligible individuals, only 7.7% are fully vaccinated [24]. Therefore, a sufficient effect of the HZ reduction due to the HZ vaccination cannot yet be observed. Overall from 2018 to 2022, we saw 661 cases of HZ in patients aged over 60 years (56% of our cases). Our analysis is limited here because we do not have information on the vaccination status of the patients. Assuming 90% vaccination, we could have prevented 547 of the 661 HZ cases in over 60-year-olds we saw from 2019 to 2022. Higher vaccination rates are urgently needed to reduce hospitalisations and prevent HZ complications[24].

Over the past 13 years, HZ was among the top 7 inpatient diagnoses in dermatology for both sexes [25, 26]. For over 80-year-olds dermatology HZ inpatients a fourfold increase from 2009 and 2022 was observed [26]. In our study, HZ(O) was the most prevalent in the 60—79 age group. Demographic change and the associated aging of the population are seen as a major reason for increasing HZ [27]. Lifetime risk of HZ increases with age and is 50% over 85 years [28]. No new naive T-cells are produced from about 40–50 years of age due to thymus involution. Consequently, CD95-positive-T-cells decrease with increasing age, so that the immune response decreases which is postulated to be a main reason for increasing HZ in aging society [27, 29]. In the future, this age group will continue to increase, so that rising HZO case numbers are to be expected [1]. Oxidative stress is known to affect HZ development. Patients with HZ exhibit lower antioxidant levels (such as uric acid, total bilirubin, and albumin) compared to those without HZ [30]. An effect of UV exposure on increased incidence of HZ has been shown [31]. This does not necessarily argue against a beneficial effect of sufficient Vitamin D levels. Oskay et al. however show no clear link between vitamin D levels and herpes zoster [30]. Overall, the data remains inconclusive. We think, it would be very helpful if future studies assessed Vitamin D levels in HZ patients and incorporated UV exposure.

Our data show HZ patients increasing primarily due to HZO patients. As the only eye hospital within 50 km, our eye centre likely treats most HZO patients. HZ at other localisations may go to peripheral hospitals. Our patient population and catchment area have not changed recently. The rise in HZO patients correlates with more eye centre treatments. Why HZO in particular is increasing is unclear. HZO tends to affect older patients, on average older than those with HZ elsewhere [32]. However, we saw an increase in all age groups in our monocentric study, which to our knowledge has not been studied before. Future analyses should detail previous diseases and immunosuppressive medication in HZO patients. Our ICD coding and billing data limited such analyses. The European consensus-based (S2k) Guideline for HZ was updated in 2016 and the German guideline in 2019, recommending inpatient admission for i.v. therapy based on consensus [14, 33]. This results in a bias in our data collection for inpatient HZO cases. However, HZO already rose before 2016/2019, so we believe the increase is real and cannot be explained by bias alone. While i.v. acyclovir may better prevent HZ encephalitis and vasculopathy, no studies directly compare i.v. and per oral (p.o.) treatment. The recommendation for i.v. therapy is consensus-based and relies on pharmacological properties and clinical findings. Oral bioavailability of acyclovir is 10—30% [34–36]. About 50% of the plasma concentration is reached in the cerebrospinal fluid [34, 36]. Therefore, oral administration results in a low concentration in the cerebrospinal fluid. Asymptomatic central nervous system involvement is common with HZ of the head and neck region, as 60% are positive for VZV in the cerebrospinal fluid [37]. This is the reason for the i.v. acyclovir recommendation. Intravenous acyclovir therapy should better prevent zoster encephalitis and vasculopathy. This recommendation is not based on studies comparing i.v. therapy vs. p.o. therapy. Valaciclovir, a prodrug of aciclovir, offers a 54% bioavailability [38]. One study compared p.o. administration of both drugs and found no advantage regarding post-zoster neuralgia or acute retinal necrosis for either drug [39]. Head-to-head studies comparing p.o. valaciclovir therapy with i.v. aciclvoir therapy in HZ(O) are lacking. In Germany, average eye patient hospitalisation is 3.09 days [25]. HZO patients stay longer, typically 7 days. Based on the current guideline, rising HZO will substantially impact inpatient ophthalmology. Our model suggests HZO patients could double by 2040 if trends persist (Fig. 1d). Should oral valaciclovir therapy be as effective as i.v. aciclovir administration, cost savings due to lower hospitalisation rates would be possible.

In summary, we found a significant rise in HZ patients, primarily HZO. Continued increase may demand greater inpatient treatment capacity and cause greater health care costs.

## Supplementary information

Below is the link to the electronic supplementary material.

Supplementary file1 (PDF 30 KB)
