# Correction: Increasing hospitalisation of patients with herpes zoster ophthalmicus—an interdisciplinary retrospective analysis

**DOI:** 10.1007/s00417-025-06894-7

**Published:** 2025-07-26

**Authors:** Rebecca Diehl, Cornelius Wiedenmann, Thomas Reinhard, Daniel Böhringer, Franziska Schauer

**Affiliations:** 1 Department of Dermatology, Medical Center, Faculty of Medicine, University of Freiburg, Freiburg, Germany; 2 Eye Center, Medical Center, Faculty of Medicine, University of Freiburg, Freiburg, Germany

**Correction: Graefe's Archive for Clinical and Experimental Ophthalmology (2023) 262:583-588**

10.1007/s00417-023-06277-w

Upon re-evaluation of our data, we identified that some patients were erroneously counted multiple times when they received treatment across different years. This misrepresentation led to an overestimation of case numbers in our original analysis. To rectify this error, we have now considered only the **first recorded appearance of each patient **diagnosed with herpes zoster (HZ) or herpes zoster ophthalmicus (HZO), ensuring that follow-up treatments were not mistakenly included as new cases.

This correction has resulted in a decrease in the total patient count from **3,034 to 2,829 patients**, with 1,480 female and 1,349 male patients. Patient distribution was updated as follows: dermatology department treated 1,839 patients (reduced from 1,879), the eye centre treated 869 cases (reduced from 920), and 121 patients presented in both departments (reduced from 235). All demographic data including median ages and sex distribution have been recalculated based on the corrected cohort. The only relevant demographic change was the median age for men, which was newly calculated as 62 years instead of the previously reported 63 years.

**Statistical Analyses:** All figures and statistical analyses have been revised to reflect the accurate patient count. Figure 1 shows: (a) the share of HZO patients in total HZ patients at both hospitals, (b) HZO treatment according to specialty, (c) HZO patients sorted by age from 2009-2022, and (d) HZO patients at both hospitals from 2009 to 2022 with predicted cases assuming stable growth. Figure 2 displays: (a) age distribution with median of patients with HZO and HZ of other localizations, and (b) gender distribution for HZ other localizations and HZO. Supplement Figure 1 has been revised to show: (a) inpatient treatment from 2009-2022 in ophthalmology, (b) inpatient treatment from 2009-2022 in dermatology, (c) outpatient treatment from 2009-2022 in ophthalmology, and (d) outpatient treatment from 2009-2022 in dermatology. Supplement Figure 2 has been updated to display: (a) HZ patients with localizations other than HZO sorted by age from 2009-2022, and (b) catchment area by specialtyThe linear regression analysis for HZO patients per year remains highly significant (*p* < 0.05) with variance of 0.9143, and the projection for doubling of HZO cases by 2040 remains valid based on the corrected data (Fig. 1d). Figure 2 displays: (a) age distribution with median of patients with HZO and HZ of other localizations, and (b) gender distribution for HZ other localizations and HZO. The vaccination impact analysis was updated to reflect that from 2018 to 2022, we observed 739 cases of HZ in patients aged 60 years and older, representing 55% of our cases. Assuming 90% vaccination efficacy, we estimate that 665 of these 739 HZ cases in patients over 60 could have been prevented.

**Fig. 1:**
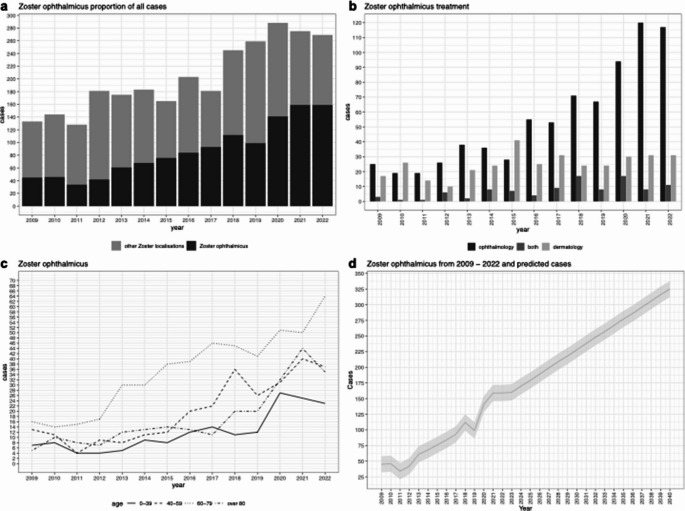
**a** Share of HZO patients in total HZ patients at both hospitals. **b** HZO treatment according to speciality. **c** HZO patients sorted by age from 2009 - 2022, **d** HZO patients at both hospitals from 2009 to 2022 and predicted cases assumed at stable growth

**Fig. 2:**
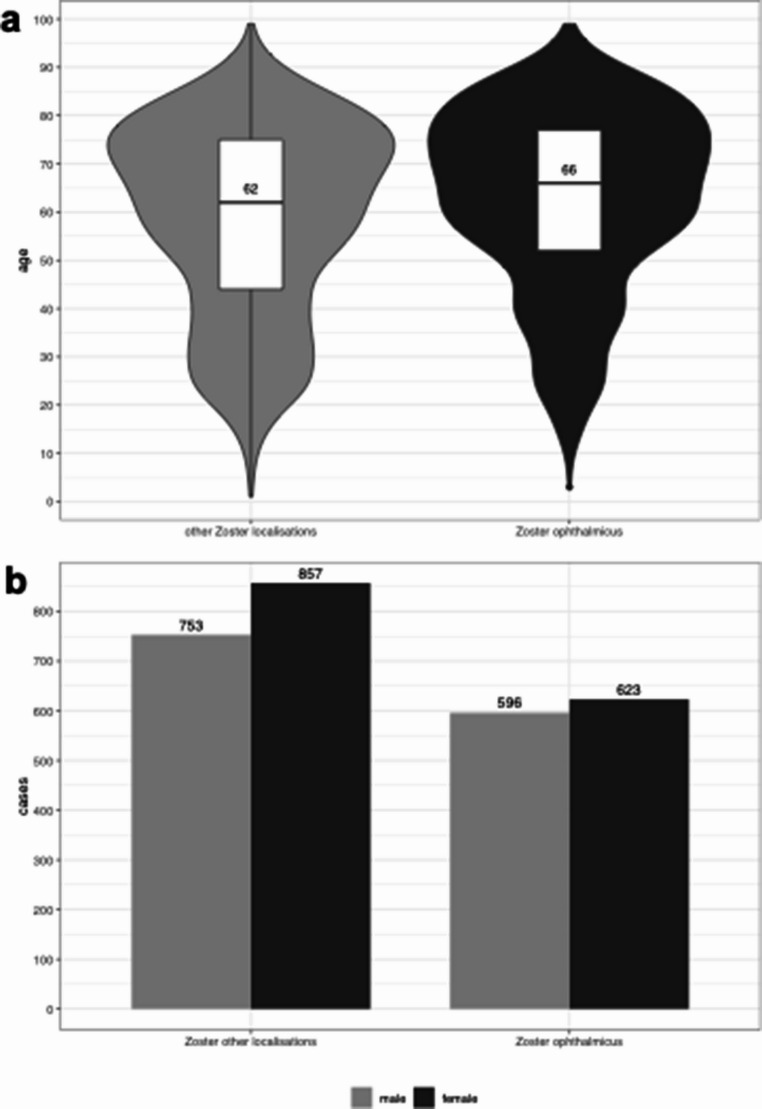
**a** Age distribution with median of patients with HZO and HZ of other localisations. **b** Gender distribution HZ other localisations and HZO

## Statement of Impact

Despite this correction, we emphasize that **the main conclusions and context of the study remain unchanged**. The observed trends in herpes zoster ophthalmicus incidence, age distribution patterns, and treatment location preferences are consistent with our original findings. The statistical significance of our key observations, including the increase in HZO patients across all age groups and the projected doubling of cases by 2040, remains robust.

## Supplementary Information

Below is the link to the electronic supplementary material.

Supplementary file1 (DOCX 216 KB)

