# Supplementary material for: Increasing hospitalisation of patients with herpes zoster ophthalmicus—an interdisciplinary retrospective analysis
Source: Graefes Arch Clin Exp Ophthalmol. 2023 Oct 20;262(2):583–8. doi: 10.1007/s00417-023-06277-w (PMC10844404; doi:10.1007/s00417-023-06277-w)
Supplement: Supplementary file 1 — Supplementary file1 (PDF 30 KB) [file 417_2023_6277_MOESM1_ESM.pdf]

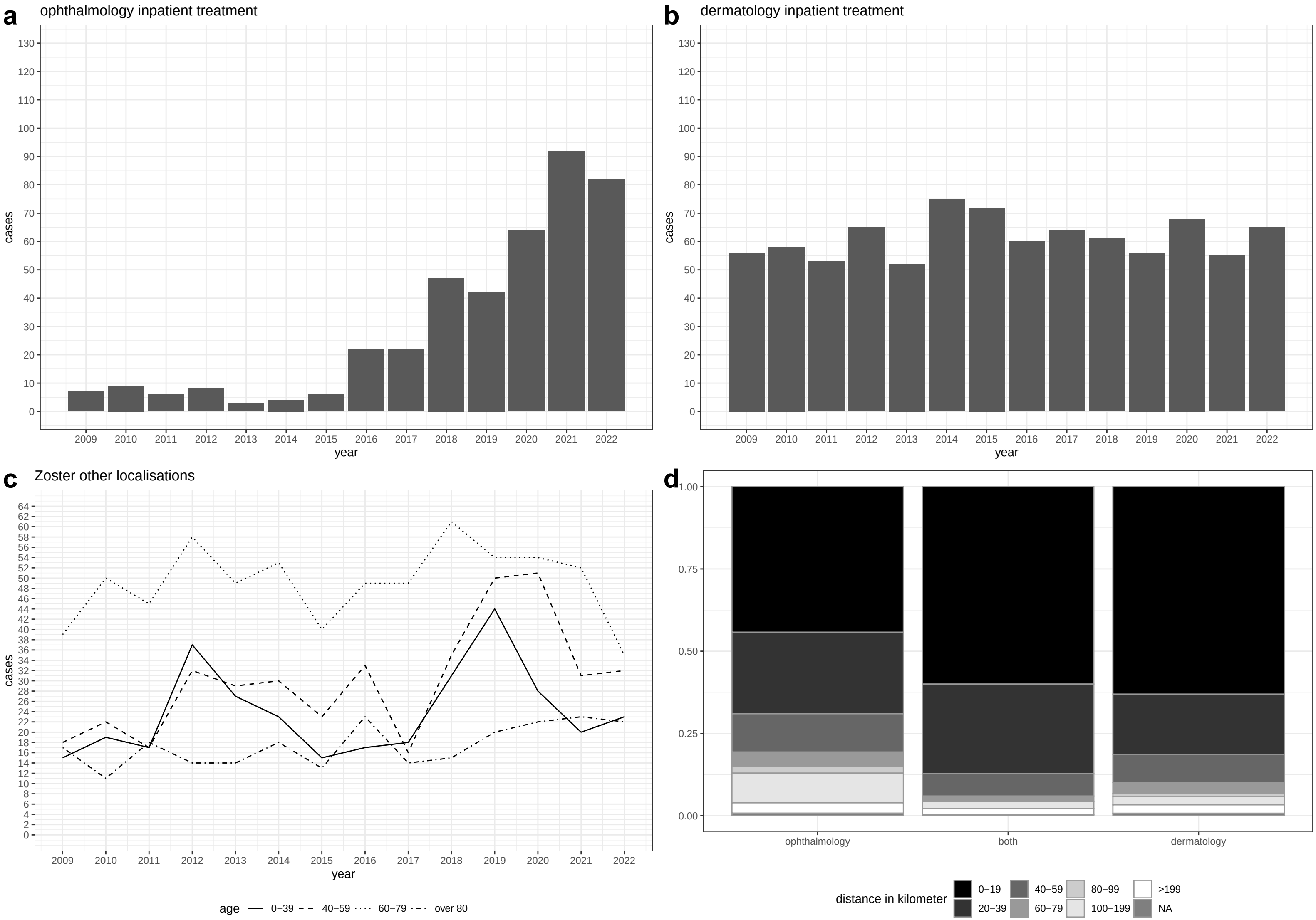

a
ophthalmology inpatient treatment
b
dermatology inpatient treatment
cases
year
2009 2010 2011 2012 2013 2014 2015 2016 2017 2018 2019 2020 2021 2022
0 10 20 30 40 50 60 70 80 90 100 110 120 130
c
Zoster other localisations
age 0−39 40−59 60−79 over 80
d
0.00 0.25 0.50 0.75 1.00
ophthalmology
both
dermatology
distance in kilometer
0−19
20−39
40−59
60−79
80−99
100−199
>199
NA
