# Supplementary material for: Correction: Increasing hospitalisation of patients with herpes zoster ophthalmicus—an interdisciplinary retrospective analysis
Source: Graefes Arch Clin Exp Ophthalmol. 2025 Jul 26;263(11):3277–9. doi: 10.1007/s00417-025-06894-7 (PMC12675612; doi:10.1007/s00417-025-06894-7)
Supplement: Supplementary file 1 — Supplementary file1 (DOCX 216 KB) [file 417_2025_6894_MOESM1_ESM.docx]

**Supplement Fig. 1** **a** Inpatient treatment from 2009 – 2022 in ophthalmology, **b** Inpatient treatment from 2009 – 2022 in dermatology, **c** Outpatient treatment from 2009 – 2022 in ophthalmology, **d** Outpatient treatment from 2009 – 2022 in dermatology

**Supplement Fig. 2 a** HZ patients other localisations then HZO sorted by age from 2009 – 2022, **b** Catchment area by speciality
